# Supplementary material for: Mammalian copper homeostasis requires retromer-dependent recycling of the high-affinity copper transporter 1
Source: J Cell Sci. 2020 Aug 25;133(16):jcs249201. doi: 10.1242/jcs.249201 (PMC7473646; doi:10.1242/jcs.249201)
Supplement: Reviewer comments [file joces-133-249201_review_history.pdf]

Mammalian copper homeostasis requires retromer-dependent recycling of the high-affinity copper transporter 1

Rachel Curnock and Peter J Cullen
DOI: 10.1242/jcs.249201

Editor: Daniel Billadeau

Review timeline

Original submission:	27 May 2020
Editorial decision:	25 June 2020
First revision received:	16 July 2020
Accepted:	20 July 2020

Original submission

Decision letter

MS ID#: JOCES/2020/249201
MS TITLE: Mammalian copper homeostasis requires retromer-dependent recycling of the high-affinity copper transporter 1 (CTR1/SLC31A1)
AUTHORS: Rachel Curnock and Peter J Cullen
ARTICLE TYPE: Research Article

We have now reached a decision on the above manuscript.

To see the reviewers' reports and a copy of this decision letter, please go to: https://submit-jcs.biologists.org and click on the 'Manuscripts with Decisions' queue in the Author Area. (Corresponding author only has access to reviews.)

As you will see, the reviewers have some concerns that prevent me from accepting the paper at this stage. After reading the reviews, I believe that you should be able to easily address them. They suggest, however, that a revised version might prove acceptable, if you can address their concerns. If you think that you can deal satisfactorily with the criticisms on revision, I would be pleased to see a revised manuscript. We would then return it to the reviewers.

We are aware that you may currently be unable to access the lab to undertake experimental revisions. If it would be helpful, we encourage you to contact us to discuss your revision in greater detail. Please send us a point-by-point response indicating where you are able to address concerns raised (either experimentally or by changes to the text) and where you will not be able to do so within the normal timeframe of a revision. We will then provide further guidance. Please also note that we are happy to extend revision timeframes as necessary.

Please ensure that you clearly highlight all changes made in the revised manuscript. Please avoid using 'Tracked changes' in Word files as these are lost in PDF conversion.

I should be grateful if you would also provide a point-by-point response detailing how you have dealt with the points raised by the reviewers in the 'Response to Reviewers' box. Please attend to all of the reviewers' comments. If you do not agree with any of their criticisms or suggestions please explain clearly why this is so.

© 2020. Published by The Company of Biologists under the terms of the Creative Commons Attribution License (https://creativecommons.org/licenses/by/4.0/).

Reviewer 1

Advance summary and potential significance to field

Mammalian copper homeostasis requires retromer-dependent recycling of the high-affinity copper transporter 1 (CTR1/SLC31A1) by Curnock & Cullen provides solid evidence of a critical sorting mechanism required for the cell surface retrieval of the copper intake transporter CTR1 in mammalian cells. The main findings are that CTR1 retrieval is dependent on retromer activity and, in contrast with ATP7A, independent of sorting nexin 27. The consequential nature of this sorting step and mechanism is documented by toxicity assays where cells lacking retromer acquire a degree of resistance to copper and platinum compound challenges.
The conclusions are robustly supported by multipronged approaches and genetic tools to modulate expression of retromer subunits such as multiple cell types rendered null buy CRISPR-Cas9 or RNAi to downregulate retromer subunits or SNX27. Data are conservatively interpreted and discussion is interesting and encompassing.
I think this manuscript will be a great addition to the copper transporter community.

Comments for the author

I have some minor suggestions that I think would enhance the paper and its readability for those outside of the endosome traffic field.

1) I would like to suggest the authors measure copper levels by ICP-MS in the retromer deficient cells in basal and copper challenged conditions as those in figure 5. This suggestion is founded on the observation that both ATP7A and CTR1 are down-regulated after retromer ablation. The down-regulation of each one of these transporters in isolation increases and decreases of cellular copper content, respectively. If CTR1 is upstream of ATP7A or if CTR1 is dominant over ATP7A activity, it predicts that copper levels may be decreased. This is the case for ATP7A, CTR1, and the COG complex, where both ATP7A and CTR1 are downregulated in COG null cells. COG null cells copper phenotype is copper depletion by the reduced CTR1 content (PMID: 28355134).

2) I suggest the retrieval of ATP7A and CTR1 are compared in what relates to the effects of SNX27 in the model figure 8.

• Minor Concerns:
o Scale Bars in Figure 1B are missing
o kDa is not listed for westerns (in methods, text or figures)
o Please specify the conditions, and antibodies used for the CTR1 internalization assays like those in figures 2 and 4.
o Provide RRDI information for antibodies
o For those not in the field, indicate the rationale of using antibodies against TrfR.

Reviewer 2

Advance summary and potential significance to field

In this manuscript the authors studied the contribution of the retromer-subunit VPS35 in the delivery of CTR1 to the cell surface. Several studies have shown that CTR1 (important copper transporter for cellular uptake of copper) can be endocytosed upon exposure of high copper. Here the authors confirmed this phenotype in different cell lines, and showed that VPS35 is required for the recycling of CTR1 back to cell surface when the excess of copper in the media was removed. The physiological importance of the sorting machinery in CTR1 functioning was assessed by treating the cells with different concentrations of copper or cisplatin (platinum-based drug). It is well known that loss of CTR1 reduces the efficacy of cisplatin.

Comments for the author

This is a focused study and clearly shows the importance of retromer in CTR1 delivery from endosomes to the cell surface. I have only minor comments.

© 2020. Published by The Company of Biologists under the terms of the Creative Commons Attribution License (https://creativecommons.org/licenses/by/4.0/).

In figure 1 the authors show that loss of VPS35 reduces the amount of CTR1 at the cell surface, but the total amount was not shown. I expected that the total amount is also reduced, can the authors confirm this? In case the total amount is reduced is this because of increased lysosomal degradation (see also figure 2).

In figure 5 the authors suggest that internalized-CTR1 is sequestered in VPS35-positive compartments upon copper exposure, and is released and subsequently delivered to the plasma membrane when the copper levels drop to normal physiological levels. How sure are the authors that CTR1 has been recycled? Could it be that another pool of CTR1 (partly newly synthesized) is transported to the plasma membrane?
It would be important to assess whether the total amount of CTR1 is not affected by high copper exposure and stay identical upon the washout experiment. This to rule out that copper exposure does not increase the lysosomal degradation of CTR1. If this latter is the case it would suggest that another mechanism would also be involved?

First revision

Author response to reviewers' comments

Reviewer 1:

Mammalian copper homeostasis requires retromer-dependent recycling of the high-affinity copper transporter 1 (CTR1/SLC31A1) by Curnock & Cullen provides solid evidence of a critical sorting mechanism required for the cell surface retrieval of the copper intake transporter CTR1 in mammalian cells. The main findings are that CTR1 retrieval is dependent on retromer activity and, in contrast with ATP7A, independent of sorting nexin 27. The consequential nature of this sorting step and mechanism is documented by toxicity assays where cells lacking retromer acquire a degree of resistance to copper and platinum compound challenges. The conclusions are robustly supported by multipronged approaches and genetic tools to modulate expression of retromer subunits such as multiple cell types rendered null buy CRISPR-Cas9 or RNAi to downregulate retromer subunits or SNX27. Data are conservatively interpreted and discussion is interesting and encompassing. I think this manuscript will be a great addition to the copper transporter community.

We thank the reviewer for their kind and positive summary.

Reviewer 1 Comments for the Author:
I have some minor suggestions that I think would enhance the paper and its readability for those outside of the endosome traffic field.

1) I would like to suggest the authors measure copper levels by ICP-MS in the retromer deficient cells in basal and copper challenged conditions as those in figure 5. This suggestion is founded on the observation that both ATP7A and CTR1 are down-regulated after retromer ablation. The down-regulation of each one of these transporters in isolation increases and decreases of cellular copper content, respectively. If CTR1 is upstream of ATP7A or if CTR1 is dominant over ATP7A activity, it predicts that copper levels may be decreased. This is the case for ATP7A, CTR1, and the COG complex, where both ATP7A and CTR1 are down- regulated in COG null cells. COG null cells copper phenotype is copper depletion by the reduced CTR1 content (PMID: 28355134).

This is an excellent suggestion. Unfortunately, we do not currently have access to the technology required to perform these experiments. Moreover, the ongoing restriction imposed by the COVID-19 pandemic severely limit our ability to establish a new collaboration in order to perform these experiments in a timely manner.

2) I suggest the retrieval of ATP7A and CTR1 are compared in what relates to the effects of

© 2020. Published by The Company of Biologists under the terms of the Creative Commons Attribution License (https://creativecommons.org/licenses/by/4.0/).

SNX27 in the model figure 8.

We have revised Figure 8 to include the SNX27-retromer mediated sorting of ATP7A and expanded the figure legend appropriately.

Minor Concerns:
Scale Bars in Figure 1B are missing. **Apologies, these have now been added.**

kDa is not listed for westerns (in methods, text or figures). **Apologies, these have now been added.**

Please specify the conditions, and antibodies used for the CTR1 internalization assays like those in figures 2 and 4. **This information has now been added.**

Provide RRDI information for antibodies. **This has been added.**

For those not in the field, indicate the rationale of using antibodies against TrfR. **A statement clarifying this point has been added to the figure legend.**

Reviewer 2:

In this manuscript the authors studied the contribution of the retromer-subunit VPS35 in the delivery of CTR1 to the cell surface. Several studies have shown that CTR1 (important copper transporter for cellular uptake of copper) can be endocytosed upon exposure of high copper. Here the authors confirmed this phenotype in different cell lines, and showed that VPS35 is required for the recycling of CTR1 back to cell surface when the excess of copper in the media was removed. The physiological importance of the sorting machinery in CTR1 functioning was assessed by treating the cells with different concentrations of copper or cisplatin (platinum- based drug). It is well known that loss of CTR1 reduces the efficacy of cisplatin.

Reviewer 2 Comments for the Author:
This is a focused study and clearly shows the importance of retromer in CTR1 delivery from endosomes to the cell surface. I have only minor comments.

We thank the reviewer for their positive summary.

In figure 1 the authors show that loss of VPS35 reduces the amount of CTR1 at the cell surface, but the total amount was not shown. I expected that the total amount is also reduced, can the authors confirm this? In case the total amount is reduced is this because of increased lysosomal degradation (see also figure 2).

Under retromer suppression CTR1 total levels are not reduced, see for example Figure 3B and Figure 7B. The active glycosylated full-length cell surface form of CTR1 is retromer-dependent whereas the truncated non-glycosylated form which is predominant in the total cell lysate is unaffected by retromer depletion. The immunofluorescence signal in Figure 2 is specific to the trafficking of cell surface CTR1; here the CTR1 antibody was surface bound on ice and then underwent internalisation in the presence of copper at 37°C. Therefore, like the surface biotinylation data, the immunofluorescence data specifically highlights the role of retromer in regulating the trafficking of the glycosylated full-length cell surface CTR1.

In figure 5 the authors suggest that internalized-CTR1 is sequestered in VPS35-positive compartments upon copper exposure, and is released and subsequently delivered to the plasma membrane when the copper levels drop to normal physiological levels. How sure are the authors that CTR1 has been recycled? Could it be that another pool of CTR1 (partly newly synthesized) is transported to the plasma membrane? It would be important to assess whether the total amount of CTR1 is not affected by high copper exposure and stay identical upon the washout experiment. This to rule out that copper exposure does not increase the lysosomal degradation of CTR1. If this latter is the case it would suggest that another mechanism would also be involved?

© 2020. Published by The Company of Biologists under the terms of the Creative Commons Attribution License (https://creativecommons.org/licenses/by/4.0/).

Given the complexities of intracellular integral protein transport, we cannot definitively conclude that CTR1 recycling does not arise from a combination of newly synthesised CTR1 exiting the biosynthetic pathway and recycling of internalised CTR1 from endosomes. That said, the total amount of CTR1 in the washout experiments does not alter across the duration of the experiment.

<u>Second decision letter</u>

MS ID#: JOCES/2020/249201
MS TITLE: Mammalian copper homeostasis requires retromer-dependent recycling of the high-affinity copper transporter 1
AUTHORS: Rachel Curnock and Peter J Cullen
ARTICLE TYPE: Research Article

I am happy to tell you that your manuscript has been accepted for publication in Journal of Cell Science, pending standard ethics checks. Where referee reports on this version are available, they are appended below.

© 2020. Published by The Company of Biologists under the terms of the Creative Commons Attribution License (https://creativecommons.org/licenses/by/4.0/).
